# Nitrogen-Doped Carbon Dots Encapsulated a Polyoxomolybdate-Based Coordination Polymer as a Sensitive Platform for Trace Tetracycline Determination in Water

**DOI:** 10.3390/nano13192676

**Published:** 2023-09-29

**Authors:** Yanqiu Zhang, Minrui Sun, Yang Lu, Mingguo Peng, Erdeng Du, Xia Xu

**Affiliations:** 1 School of Environmental Science and Engineering, Changzhou University, Changzhou 213164, China; 2 School of Urban Construction, Changzhou University, Changzhou 213164, China

**Keywords:** tetracycline, fluorescent sensing, metal–organic coordination polymer, nitrogen-doped carbon

## Abstract

The requirement of simple, efficient and accurate detection of tetracycline (TC) in water environments poses new challenges for sensing platform development. Here, we report a simple method for TC sensing via fluorescence detection based on metal–organic coordination polymers (MOCPs, (4-Hap)_4_(Mo_8_O_26_)) coated with nitrogen-doped carbon dots (NCDs). These NCDs@(4-Hap)_4_(Mo_8_O_26_) composites showed excellent luminescence features of NCDs with stable bright-blue emission under UV light. The results of the sensing experiment showed that the fluorescence of NCDs@(4-Hap)_4_(Mo_8_O_26_) can be quenched by TC (166 µM) with 94.1% quenching efficiency via the inner filter effect (IFE) in a short time (10 s), with a detection limit (LOD) of 33.9 nM in a linear range of 8–107 µM. More significantly, NCDs@(4-Hap)_4_(Mo_8_O_26_) showed a high selectivity for TC sensing in the presence of anions and metal cations commonly found in water environments and can be reused in at least six cycles after washing with alcohol. The potential practicality of NCDs@(4-Hap)_4_(Mo_8_O_26_) was verified by sensing TC in real water samples with the standard addition method, and satisfactory recoveries from 91.95% to 104.72% were obtained.

## 1. Introduction

Because of its advantages of low cost and effective treatment for bacterial pathogens, antibiotics are widely used in agriculture, animal husbandry, aquaculture and other fields [1]. It is estimated that the global use of antibiotics is expected to reach 225,000 tons by 2020 [2], of which China is the world’s largest user, accounting for about 45% of global consumption each year [3]. However, studies have shown that about 30–70% of antibiotics are not metabolized sufficiently by humans and animals and thus released into the environment in their original form. In recent years, the presence of antibiotics has been found in surface waters in East Asia, Southeast Asia, the United States, Europe and other places at concentrations between ng·L^−1^ and mg·L^−1^ [4,5]. Despite the low concentration, long-term exposure to antibiotics is harmful to microbial, plant, animal and human health. Based on the investigation of the U.S. Food and Drug Administration, tetracycline (TC) is one of the most commonly used antibiotics in veterinary treatment and animal growth [6]. Considering that the long-term existence of TC in the environment may pose potential risks to organisms such as endocrine disruption, neurotoxicity and genotoxicity, the accurate and quick identification of the concentration of TC in water environments is imperative.

For more effective monitoring of TC in water environments, a number of analytical techniques have been developed in recent years [7,8,9]. As the fluorescent sensing method can avoid the complex operation of traditional large-scale instrumental detection methods, the detection of antibiotics with the fluorescence sensing method has attracted increasing attention recently. Usually, the fluorescence method is based on materials including rare earth metals, fluorescent dyes and some quantum dots [10,11]. However, such fluorescent molecules usually have small sizes and high solubility, which make them difficult to recover in aqueous environments.

Metal–organic coordination polymers (MOCPs) composed of metal ions and organic ligands have attracted much attention due to their diverse structures and abundant active sites [12]. The unique structure and properties give MOCPs broad application prospects in the fields of catalysis [13,14,15], drug delivery [16,17], adsorption [18,19], gas storage [20,21] and sensing [22,23]. At present, there are studies on the application of MOCPs in the field of TC detection. Wang’s group studied the luminescence performance of ZIF-8 and realized the detection of TC in water by ZIF-8 based on the aggregation-induced emission effect [24]. Li’s group fabricated a Zn(bix) coordination polymer (bix = 1,4-bis(imidazol-1-ylmethyl)benzene) and achieved the quantitative detection of TC based on the luminescence of Zn(bix) turned on [25]. However, the intrinsic luminescence of MOCPs usually comes from the charge transfer between the metal and the ligand, or from the luminescence of the metal and organic ligand, which often has low quantum yields and weak luminescence, thus limiting their further practical application. The post-synthetic modification (PSM) method can enhance the luminescence properties of the original MOCPs by loading fluorescence functional materials while retaining the properties of the original MOCPs. In recent years, carbon dots (CDs), a kind of fluorescence material with high quantum yield and long lifetime, have attracted wide attention due to their strong chemical inertness and low toxicity, making them ideal luminescent guests to improve the luminescence performance of MOCPs.

In this study, a simple strategy at room temperature was used to fabricate a fluorescence sensor (NCDs@(4-Hap)_4_(Mo_8_O_26_)) by introducing nitrogen-doped carbon dots (NCDs) onto a polyoxomolybdate-based coordination polymer ((4-Hap)_4_(Mo_8_O_26_)) for efficient, sensitive and selective detection of TC in aqueous solution. NCDs@(4-Hap)_4_(Mo_8_O_26_) exhibits bright-blue fluorescence emission derived from the NCDs and can be quenched with the addition of TC, which is caused by the inner filter effect (IFE). In addition, NCDs@(4-Hap)_4_(Mo_8_O_26_) can achieve quantitative detection of TC in a wide linear range (8–107 µM) with a detection limit (LOD) of 33.9 nM. Significantly, NCDs@(4-Hap)_4_(Mo_8_O_26_) has been successfully used in sensing TC in real water samples (i.e., river water and tap water), suggesting great practical application potential in the detection of water-environment contaminants.

## 2. Experiment and Method

### 2.1. Materials

All chemical reagents used in this work were analytically pure chemicals that did not require further purification. Chemicals used for (4-Hap)_4_(Mo_8_O_26_) preparation, namely, ammonium molybdate tetrahydrate, cadmium chloride hemi(pentahydrate), 4-aminopyridine and antibiotics including tetracycline (TC), ciprofloxacin (CIP), chloramphenicol (CAP), sulfadiazine (SDZ), sulfamethazine (SMZ), thiamphenicol (THI), metronidazole (MDZ) and ornidazole (ODZ), were purchased from Aladdin Industrial Co., Ltd. (Shanghai, China). The other chemicals used in this work were purchased from Sinopharm Chemical Reagent Co., Ltd. (Beijing, China).

### 2.2. Instrumentation

The microstructure was determined by a scanning electron microscope (SEM), JSM-IT500HR, Tokyo, Japan. Powder X-ray diffraction (XRD) patterns were examined on a Bruker D8 X-ray diffractometer (Billerica, MA, USA) with CuKα line as radiation source. Fourier-transform infrared (FTIR) spectra were recorded on a Thermo Scientific Nicolet iS 5, Waltham, MA, USA. X-ray photoelectron spectra (XPS) were measured by a Thermo Scientific K-Alpha, Waltham, MA, USA to determine the surface element composition of the samples. The fluorescence spectra and luminescent lifetimes were acquired on a FS5 Fluorescence Spectrometer (Edinburgh, UK), and the lifetime was obtained by the luminescent decay fitting. UV-vis absorption spectra were measured by a HACH DR-6000 UV-visible spectrophotometer (Loveland, CA, USA).

### 2.3. Fabrication of NCDs@(4-Hap)_4_(Mo_8_O_26_)

NCDs were synthesized according to a previous work [26]. In detail, 1.05 g citric acid was dispersed in 10 mL deionized H_2_O, and then, 0.335 mL ethylenediamine was added. The mixture was stirred for 30 min and then added to a 25 mL Teflon-lined autoclave and heated at 473.15 K for 5 h. After it was cooled down to room temperature, a brown-black solution was obtained and then centrifuged to remove large particles.

A hydrothermal method was used to synthesize (4-Hap)_4_(Mo_8_O_26_) according to a previous work [19]. First, 0.0685 g cadmium chloride hydrate (CdCl_2_·2.5H_2_O), 0.028 g 4-aminopyridine (4-ap, C_5_H_6_N_2_) and 0.7415 g ammonium molybdate tetrahydrate (H_24_Mo_7_N_6_O_24_·4H_2_O) were blended and added to 20 mL deionized water. Then, after 10 min ultrasound treatment, the mixture was encapsulated in a 25 mL Teflon-lined autoclave at 443 K, reacting for 72 h. After being filtered and washed with ethanol three times, (4-Hap)_4_(Mo_8_O_26_) was obtained after being dried in an oven at 60 °C for 6 h.

The prepared (4-Hap)_4_(Mo_8_O_26_) (10 mg) and NCDs (1 mL) were dispersed in 10 mL deionized water, stirring after 24 h, then centrifugated (8000 r/min) for 5 min and washed twice with methanol. After being dried at 60 °C overnight, the NCDs@(4-Hap)_4_(Mo_8_O_26_) was obtained.

### 2.4. Fluorescence Sensing of TC

The luminescence performance of NCDs@(4-Hap)_4_(Mo_8_O_26_) was investigated. First, 2 mg NCDs@(4-Hap)_4_(Mo_8_O_26_) was added to 2.5 mL deionized water, and the stability of the luminescence was confirmed by recording the fluorescence emission intensity at different time intervals over 7 days. Moreover, the fluorescence emission intensity was measured by immersing 2 mg NCDs@(4-Hap)_4_(Mo_8_O_26_) in 2.5 mL deionized water with different pH (3.0–12.0) to investigate the effects of different pH levels in aqueous solution. The response kinetics were further explored with fluorescence intensity measurement by mixing 2 mg NCDs@(4-Hap)_4_(Mo_8_O_26_) and 300 µL TC aqueous solution (1 mM) in 2.5 mL deionized water at room temperature for different incubation times.

Under optimized conditions, the sensing performance of NCDs@(4-Hap)_4_(Mo_8_O_26_) in relation to TC was studied. In detail, 2 mg NCDs@(4-Hap)_4_(Mo_8_O_26_) was dispersed in 2.5 mL deionized water, followed by adding TC aqueous solution (1 mM, 20 µL addition each time) to form solutions with different TC concentrations. After 10 s at room temperature, the emission spectra under 380 nm excitation were recorded. Afterward, the selectivity and anti-interference capability of NCDs@(4-Hap)_4_(Mo_8_O_26_) in relation to TC was further investigated. In detail, 2 mg NCDs@(4-Hap)_4_(Mo_8_O_26_) was added to 2.5 mL of solutions containing various kinds of antibiotics (100 µM, CIP, CAP, SDZ, SMZ, THI, MDZ, ODZ), anions (100 µM, F^−^, NO_2_^−^, S_2_O_3_^2−^, SO_3_^−^, HSO_3_^−^, HCO_3_^−^, HSO_4_^−^, CO_3_^2−^, NO_3_^−^, SO_4_^2−^, PO_4_^3−^, S_2_O_8_^2−^) and metal ions (100 µM, Na^+^, K^+^, Mg^2+^, Ca^2+^, Zn^2+^, Cd^2+^, Fe^2+^, Ni^2+^, Cu^2+^, Al^3+^, Ba^2+^, Fe^3+^). The fluorescence emission intensities of the solutions with and without 100 µM TC were measured separately after incubating for 10 s at room temperature.

In addition, after the detection of TC, NCDs@(4-Hap)_4_(Mo_8_O_26_) was collected, immersed in alcohol for 12 h, then washed and centrifuged for recovery. After being dried in a vacuum at 60 °C for 6 h, NCDs@(4-Hap)_4_(Mo_8_O_26_) was obtained and further reused in the detection of TC to explore the reusability.

### 2.5. Detection of TC in Environmental Samples

Environmental samples were obtained from river water in Changzhou and tap water in the laboratory of Changzhou University in Jiangsu. These collected samples were filtered with 0.45 µm pore size membrane. Then, the spiking method was used to prepared TC solution with various concentrations. The emission intensity was recorded after immersing 2 mg NCDs@(4-Hap)_4_(Mo_8_O_26_) in 2.5 mL of the above-described solutions after 10 s incubation time.

## 3. Results and Discussion

### 3.1. Characterization of Materials

**Figure 1:**
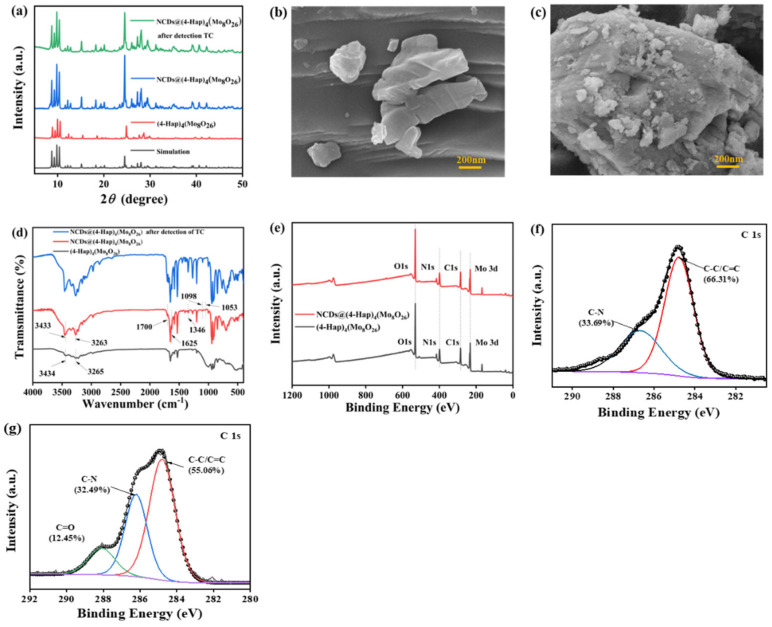
(**a**) XRD patterns of (4-Hap)_4_(Mo_8_O_26_), NCDs@(4-Hap)_4_(Mo_8_O_26_) and NCDs@(4-Hap)_4_(Mo_8_O_26_) after the detection of TC; SEM images of 4-Hap)_4_(Mo_8_O_26_) (**b**) and NCDs@(4-Hap)_4_(Mo_8_O_26_) (**c**); (**d**) FTIR spectra of (4-Hap)_4_(Mo_8_O_26_), NCDs@(4-Hap)_4_(Mo_8_O_26_) and NCDs@(4-Hap)_4_(Mo_8_O_26_) after the detection of TC; (**e**) Full range XPS of (4-Hap)_4_(Mo_8_O_26_) and NCDs@(4-Hap)_4_(Mo_8_O_26_); the C1s spectra of (4-Hap)_4_(Mo_8_O_26_) (**f**) and NCDs@(4-Hap)_4_(Mo_8_O_26_) (**g**).

The crystal structure of (4-Hap)_4_(Mo_8_O_26_) and NCDs@(4-Hap)_4_(Mo_8_O_26_) prepared in this work was characterized with XRD patterns. As described in a previous study, the basic structure of (4-Hap)_4_(Mo_8_O_26_) consisted of a dissociative β-octamolybdate anion and four discrete 4-ap ions protonated at nitrogen atom of the pyridine ring, which further construct the three-dimensional structure of (4-Hap)_4_(Mo_8_O_26_) via the hydrogen bonds. Moreover, (4-Hap)_4_(Mo_8_O_26_) demonstrates an overall negative surface charge in the pH range from 2 to 9 [19]. As depicted in Figure 1a, the major sharp diffraction peaks of (4-Hap)_4_(Mo_8_O_26_) were in line with the simulated pattern [19], indicating that the crystalline phase of (4-Hap)_4_(Mo_8_O_26_) was pure. In the XRD pattern of NCDs@(4-Hap)_4_(Mo_8_O_26_), no obvious change in peaks emerged after NCDs were loaded onto (4-Hap)_4_(Mo_8_O_26_) (Figure 1a), indicating that the framework of (4-Hap)_4_(Mo_8_O_26_) remained intact in the post-synthetic loading process. The SEM images of (4-Hap)_4_(Mo_8_O_26_) without and with loading of NCDs were captured. As illustrated in Figure 1b,c, both (4-Hap)_4_(Mo_8_O_26_) and NCDs@(4-Hap)_4_(Mo_8_O_26_) showed irregular stone shape, but compared to the pure (4-Hap)_4_(Mo_8_O_26_), NCDs@(4-Hap)_4_(Mo_8_O_26_) had a rougher surface, indicating that NCDs attached onto the surface of (4-Hap)_4_(Mo_8_O_26_). The FTIR spectra were measured to study the chemical composition of NCDs@(4-Hap)_4_(Mo_8_O_26_) (Figure 1d). In the FTIR spectra of (4-Hap)_4_(Mo_8_O_26_), the peaks in the range of 600–1000 cm^−1^ were assigned to *v* (Mo=O) and *v* (Mo-O-Mo) in β-octamolybdate, the peaks at 3265 and 3434 cm^−1^ were the non-coordinating -NH_2_ functions, the adsorption bands at 1346 and 1625 cm^−1^ related to the stretching vibration of C-N and the N-H bend vibration, respectively, and the peak at 1000 cm^−1^ was associated with C=N. In the FTIR spectra of NCDs@(4-Hap)_4_(Mo_8_O_26_), the adsorption bands of C-N and N-H were enhanced, and the bands assigned to -COO- of the NCDs at 1700, 1098 and 1053 cm^−1^ were visible, indicating the successful introduction of NCDs onto (4-Hap)_4_(Mo_8_O_26_). Moreover, the peaks associated with -NH_2_ shifted to a lower wavenumber at 3263 and 3433 cm^−1^ compared to (4-Hap)_4_(Mo_8_O_26_), and the peaks associated with -COO- shifted to a lower wavenumber than that of pristine NCDs at 1723, 1169 and 1092 cm^−1^ [27], which indicate that hydrogen bonding may be formed between -NH_2_ of (4-Hap)_4_(Mo_8_O_26_) and -COOH of NCDs. XPS was further investigated to manifest the chemical states and composition of NCDs@(4-Hap)_4_(Mo_8_O_26_). All binding energies presented in XPS were modified based on C1s of 284.8 eV. As shown in Figure 1e, four main elements, namely, C, N, O and Mo, existed in the XPS survey of both (4-Hap)_4_(Mo_8_O_26_) and NCDs@(4-Hap)_4_(Mo_8_O_26_), but the content of O was increased from 24.66 to 36.17% and the content of Mo was decreased from 8.79 to 6.06% after the induction of NCDs by (4-Hap)_4_(Mo_8_O_26_), which further confirm that the NCDs were coated on the (4-Hap)_4_(Mo_8_O_26_). In the C1s spectra of NCDs@(4-Hap)_4_(Mo_8_O_26_) (Figure 1f), the chemical bond of C=O with content of 12.45% emerged at 288.09 eV. Moreover, the content of C-C/C=C and C-N decreased from 66.31% and 33.69% to 55.08% and 32.49%, respectively, compared to that of (4-Hap)_4_(Mo_8_O_26_) (Figure 1g). These results suggest that NCDs were coated on the (4-Hap)_4_(Mo_8_O_26_) and π–π interactions may exist between NCDs and (4-Hap)_4_(Mo_8_O_26_).

### 3.2. Luminescence of NCDs@(4-Hap)_4_(Mo_8_O_26_)

**Figure 2:**
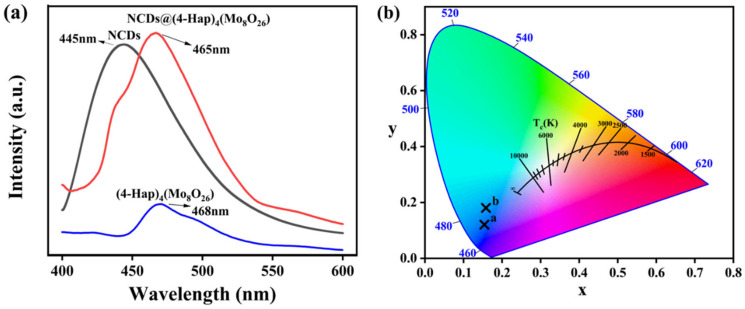
(**a**) The emission spectra of NCDs (black line, E_x_ = 365 nm), (4-Hap)_4_(Mo_8_O_26_) (blue line, E_x_ = 363 nm) and NCDs@(4-Hap)_4_(Mo_8_O_26_) (red line, E_x_ = 380 nm); (**b**) The chromaticity diagram of NCDs and NCDs@(4-Hap)_4_(Mo_8_O_26_) excited at 365 nm.

As shown in Figure S1a, (4-Hap)_4_(Mo_8_O_26_) is a light-yellow powder under natural light, and it showed a broad, weak fluorescence emission peak at 468 nm (Figure 2a) under UV light excitation (E_x_ = 363 nm). NCDs exhibited a blue fluorescence emission at 445 nm under excitation of 365 nm (Figure 2a), and situated in the blue region at point a (X = 0.15, Y = 0.12) in the CIE chromaticity diagram (Figure 2b). After loading of NCDs onto (4-Hap)_4_(Mo_8_O_26_), NCDs@(4-Hap)_4_(Mo_8_O_26_) turned brown (Figure S1b) under natural light. Similar to other quantum dots reported in the literature [28,29], the fluorescence emission of NCDs@(4-Hap)_4_(Mo_8_O_26_) exhibited a strong blue emission at 465 nm (E_x_ = 380 nm) (Figure 2a), and showed typical excitation-dependent emission, which enhanced with the excitation wavelength increased from 340 to 390 nm and gradually decreased with longer excitation wavelength (Figure S2). The strong blue fluorescence is visible to the naked eye and situated at point b (X = 0.15, Y = 0.18) in the CIE chromaticity diagram (Figure 2b). Compared to NCDs, the emission peak of NCDs@(4-Hap)_4_(Mo_8_O_26_) showed a red shift of 20 nm, indicating that the hydrogen bonds may exist between NCDs and (4-Hap)_4_(Mo_8_O_26_) [30].

As depicted in Figure S3, no obvious decline in the fluorescence emission intensity at 465 nm was observed after immersing the NCDs@(4-Hap)_4_(Mo_8_O_26_) in water for 7 days, validating the excellent fluorescence stability of NCDs@(4-Hap)_4_(Mo_8_O_26_). In addition, the fluorescence emission of NCDs@(4-Hap)_4_(Mo_8_O_26_) can be kept stable in the pH range from 3 to 12 (Figure S4), suggesting the potential application of NCDs@(4-Hap)_4_(Mo_8_O_26_) as a fluorescent sensor in water.

### 3.3. Sensing of TC

**Figure 3:**
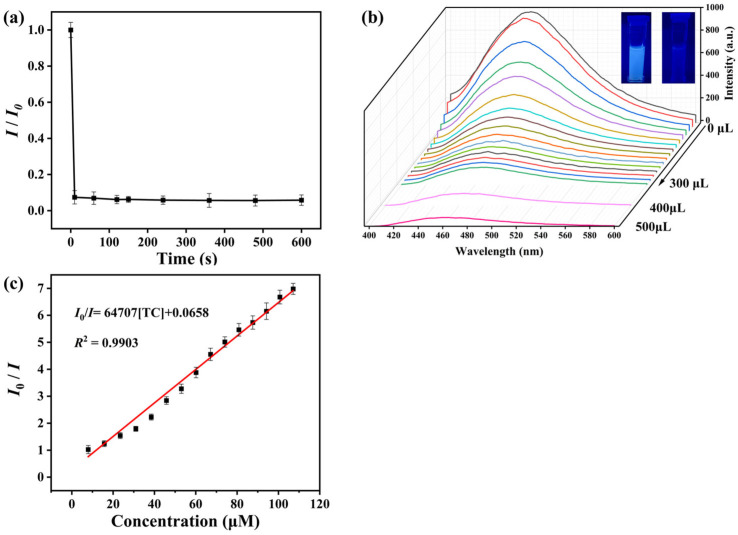
(**a**) Fluorescence intensity of the mixed solution of NCDs@(4-Hap)_4_(Mo_8_O_26_) and TC (2.5 mL, 107 µM) at 465 nm (E_x_ = 380 nm) at different times; (**b**) Fluorescent spectra (E_x_ = 380 nm) of the mixed solution of NCDs@(4-Hap)_4_(Mo_8_O_26_) and different concentrations of TC; (**c**) Calibration plot of the fluorescence intensity ratio (*I*_0_/*I*) versus concentrations of TC.

The results of the response kinetics study of NCDs@(4-Hap)_4_(Mo_8_O_26_) for TC sensing are illustrated in Figure 3a. In the study, the fluorescence was quenched in 10 s and reached equilibrium. Based on the rapid response time, all TC sensing experiments with NCDs@(4-Hap)_4_(Mo_8_O_26_) in this study were taken in real time.

Further, the performance of NCDs@(4-Hap)_4_(Mo_8_O_26_) in detecting different concentrations of TC was studied. As illustrated in Figure 3b, under excitation of 380 nm, the fluorescence emission was quenched with the addition of TC solution (1 mM) from 0 to 300 µL (20 µL increase each time). The quenching efficiency could reach 85.7% with 300 µL TC solution (1 mM) addition, and further increased to 94.1% with the 500 µL TC solution (1 mM) addition, and the blue fluorescence under UV light was visible to the naked eye from bright to dark (Figure 3b inset). The Stern–Volmer (S-V) formula [31] (Equation (1)) was used to explore the quantitative relationship between the fluorescence emission and the concentration of TC. A good linear relationship with the linear correlation coefficient (*R*^2^) of 0.9903 in an 8–107 µM concentration range was found, as shown in Figure 3c, and the *K*_sv_ value was calculated as 6.47 × 10^4^ M^−1^. According to the 3*σ* IUPAC criteria (3*σ*/slope) (*σ* is the standard deviation of three blank measurements) [32], the LOD of TC sensing was estimated to be 33.9 nM. Moreover, as shown in Table 1, the LOD for TC sensing in this work is better than most other TC-detection platforms. In addition, the performance for the detection of TC with the concentration from 0 to 8 µM, as seen in Figure S5, showed a relatively low linear correlation of 0.9299 with a *K*_sv_ value of 2.69 × 10^4^ M^−1^, indicating a higher LOD in practical sensing application than the calculated LOD of 33.9 nM, which may be due to the large error in the detection of trace TC in water.

*I*_0_/*I* = *K*_sv_[M] + 1 (1)

where *I*_0_ and *I* are the emission intensities of NCDs@(4-Hap)_4_(Mo_8_O_26_) before and after the detection of TC, respectively; *K*_sv_ represents the S-V constant (M^−1^); [M] is the molar concentration of TC (µM).

### 3.4. Selectivity and Anti-Interference

**Figure 4:**
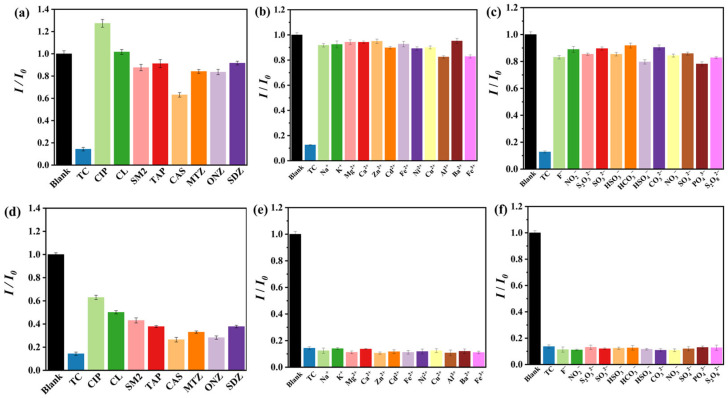
Fluorescence intensities at 465 nm of NCDs@(4-Hap)_4_(Mo_8_O_26_) in different (**a**) antibiotics (100 µM), (**b**) metal ions (100 µM) and (**c**) anions (100 µM) in aqueous solutions; fluorescence intensities of NCDs@(4-Hap)_4_(Mo_8_O_26_) (E_x_ = 380 nm) at 465 nm with the coexistence of TC (100 µM) and different (**d**) antibiotics (100 µM), (**e**) metal ions (100 µM) and (**f**) anions (100 µM) in aqueous solutions.

The current bottleneck that limits the application of chemical sensors in real water environments is the fact that the fluorescence signal can be interfered with by other species. Thus, the selectivity and anti-interference experiments were conducted with other antibiotics and common ions in a water environment. As depicted in Figure 4a–c, with the addition of TC and other selected species in NCDs@(4-Hap)_4_(Mo_8_O_26_) suspension, only TC quenched the fluorescence of NCDs@(4-Hap)_4_(Mo_8_O_26_), whereas other antibiotics and the studied ions showed little effect on the fluorescence emission of NCDs@(4-Hap)_4_(Mo_8_O_26_), suggesting the high selectivity of NCDs@(4-Hap)_4_(Mo_8_O_26_) for TC sensing. Moreover, the other antibiotics and common ions were mixed with TC and added to the NCDs@(4-Hap)_4_(Mo_8_O_26_) suspension; then, the fluorescence of the mixed solution was monitored. The results in Figure 4d–f show that TC can still quench the fluorescence of NCDs@(4-Hap)_4_(Mo_8_O_26_) with similar quenching efficiency in the presence of the other antibiotics and common ions, suggesting excellent anti-interference of NCDs@(4-Hap)_4_(Mo_8_O_26_) as a TC sensor.

### 3.5. Mechanism of NCDs@(4-Hap)_4_(Mo_8_O_26_) for TC Detection

**Figure 5:**
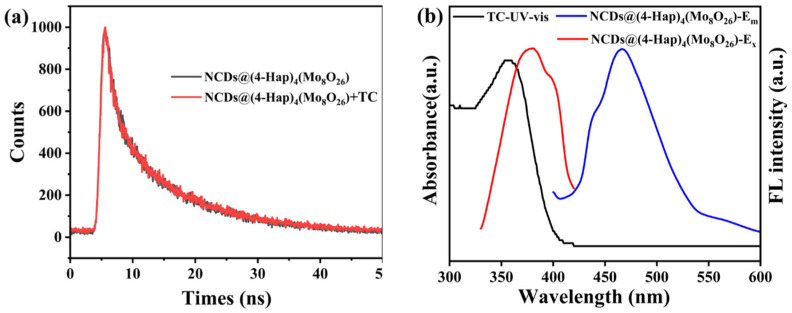
(**a**) The fluorescence decay curve of NCDs@(4-Hap)_4_(Mo_8_O_26_) (black line) and NCDs@(4-Hap)_4_(Mo_8_O_26_) after detection of TC (red line); (**b**) The UV-vis adsorption spectra of TC (black line), and the excitation spectra (red line, E_m_ = 465 nm) and emission spectra (blue line, E_x_ = 380 nm) of NCDs@(4-Hap)_4_(Mo_8_O_26_).

Studies have shown that the reasons for fluorescence quenching of sensing materials caused by the detected substance mainly include the following aspects: (i) The target detection substance interacts with the sensing material, resulting in the structural collapse of the sensing material [40]. (ii) The organic ligands or metal ions in the sensing material react with the target detection substance, destroying the original energy transfer [41]. (iii) The UV-vis spectrum of the target detection substance overlaps with the fluorescence excitation spectrum of the sensing material, resulting in fluorescence quenching by IFE [42]. To investigate the mechanism, the structure of NCDs@(4-Hap)_4_(Mo_8_O_26_) was first explored. As depicted in Figure 1a, compared to the XRD patterns of the original NCDs@(4-Hap)_4_(Mo_8_O_26_), there was no significant change in NCDs@(4-Hap)_4_(Mo_8_O_26_) after sensing TC, indicating that the structure remained intact; thus, the fluorescence quenching caused by structural damage was excluded. Since the resonance energy transfer during the quenching process can lead to a significant decrease in the fluorescence lifetime, the lifetime of NCDs@(4-Hap)_4_(Mo_8_O_26_) in aqueous solutions before and after sensing TC was investigated. As illustrated in Figure 5a, the average lifetime of the NCDs@(4-Hap)_4_(Mo_8_O_26_) before and after the detection of TC was 10.86 ns and 10.58 ns, respectively, which remained stable, indicating that the quenching process is static and the resonance energy transfer is not the dominant reason for the fluorescence quenching. Further, the UV-vis absorption spectra of TC and the fluorescence excitation/emission spectrum of NCDs@(4-Hap)_4_(Mo_8_O_26_) were recorded. As shown in Figure 5b, the UV-vis absorption spectra of TC overlaps with the excitation spectrum of NCDs@(4-Hap)_4_(Mo_8_O_26_), which means that some UV light was absorbed by TC and shielded the excitation light of NCDs@(4-Hap)_4_(Mo_8_O_26_), causing the fluorescence to be quenched through the IFE, which usually shows a higher selectivity and shorter response time than other mechanisms [43]. Moreover, TC molecules existed in four distinct species at different pH levels. At a pH lower than 3.3 and in a pH range from 3.3 to 7.7, the dominant species of TC was TC cationic species (TCH^3+^) and the nearly neutral or zwitterionic species (TCH^2±^), respectively, which leads to favorable electrostatic attractions between TC and (4-Hap)_4_(Mo_8_O_26_) with a negatively charged surface. This can pre-concentrate TC on NCDs@(4-Hap)_4_(Mo_8_O_26_) and thereby improve the sensitivity and shorten the response time of TC detection. Additionally, no new adsorption peak on the FTIR spectra of NCDs@(4-Hap)_4_(Mo_8_O_26_) was observed after the detection of TC, indicating no chemical interactions between NCDs@(4-Hap)_4_(Mo_8_O_26_) and TC. The above speculation was evidenced by the good recyclability of NCDs@(4-Hap)_4_(Mo_8_O_26_), as shown in Figure S6, which could be reused in the next six runs after it was washed with alcohol.

### 3.6. Sensing TC in Environmental Samples

The feasibility of NCDs@(4-Hap)_4_(Mo_8_O_26_) used for sensing TC in environmental water samples was investigated. Two samples, one from tap water and the other from river water, were used as the solvent to prepare TC solutions with various concentrations based on the standard addition method. Recoveries of NCDs@(4-Hap)_4_(Mo_8_O_26_) for TC sensing were calculated by Equation (2).

Recovery = *C*_d_/*C*_s_ × 100% (2)

where *C*_d_ (mg·L^−1^) represents the detected concentration of TC, and *C*_s_ (mg·L^−1^) is the spiked concentration of TC.

As shown in Table 2, the recoveries were obtained from 91.95% to 104.72%, and the relative standard deviation (*RSD*) were calculated to be in the range from 1.24% to 5.93%, suggesting the accuracy and reliability of NCDs@(4-Hap)_4_(Mo_8_O_26_) in TC sensing in water environments.

## 4. Conclusions

In conclusion, NCDs@(4-Hap)_4_(Mo_8_O_26_) with satisfactory fluorescence performance for sensitivity and selective sensing of the trace amount of TC in aqueous solutions was fabricated via post-synthetic modification of NCDs on (4-Hap)_4_(Mo_8_O_26_). NCDs were successfully coated on (4-Hap)_4_(Mo_8_O_26_) and provide a strong and stable blue emission to NCDs@(4-Hap)_4_(Mo_8_O_26_). NCDs@(4-Hap)_4_(Mo_8_O_26_) can be used for sensing TC with fluorescence quenching in 10 s, and the LOD can reach 33.9 nM with a linear range in 8–107 µM. In particular, the change of the fluorescent signal from bright blue to dark blue under UV light is visible to the naked eye, making NCDs@(4-Hap)_4_(Mo_8_O_26_) more practical in TC sensing. The potential practical application was further verified by the standard addition experiments with good recoveries in real water samples. In addition, mechanism studies showed that the fluorescence quenching may be attributed to the inner filter effect, which usually provides high sensitivity and quick response for sensing. It is believed that this strategy can provide a new viewpoint in constructing reliable methods for TC sensing in water environments.

## Figures and Tables

**Table 1:** Comparison of the fluorescence-sensing performance for sensing TC.

Material	LOD (nM)	Linear Range (µM)	Ref
GUCDs	165	0.5–25	[33]
NH_2_-MIL-53(Al)	920	1.5–70	[34]
PCN-128Y	30	0–1	[35]
Europium-doped carbon dots	15.8	0–623.8	[36]
BSA-AuNCs	65	0.2–10	[37]
N,S-doped carbon nanodots	160	0.8–10	[38]
Nitrogen-doped durian shell-derived carbon dots	75	0–30	[39]
NCDs@(4-Hap)_4_(Mo_8_O_26_)	33.9	8–107	This work

**Table 2:** Determination of TC in environmental water samples.

Samples	Spiked Concentration (µM)	Measured (µM)	Recovery (%)	*RSD* (%)
River water	25	23.1	92.2	5.73
	50	48.5	96.9	2.22
	100	98.3	98.3	1.24
Tap water	25	23.0	92.0	5.93
	50	47.5	94.9	3.69
	100	104	105	3.26

## Data Availability

Data will be made available on request.

## Supplementary Materials

The following supporting information can be downloaded at https://www.mdpi.com/article/10.3390/nano13192676/s1: Figure S1: Photographs of (a) (4-Hap)_4_(Mo_8_O_26_), (b) NCDs@(4-Hap)_4_(Mo_8_O_26_) under natural light; Figure S2: Emission spectra for NCDs@(4-Hap)_4_(Mo_8_O_26_) in water at different wavelengths; Figure S3: Relative fluorescence intensity of NCDs@(4-Hap)_4_(Mo_8_O_26_) with excitation at 380 nm in 7 days; Figure S4: The relative fluorescence intensity of NCDs@(4-Hap)_4_(Mo_8_O_26_) in water under different pH levels (E_x_ = 380 nm); Figure S5: Calibration plot of the fluorescence intensity ratio (*I*_0_/*I*) versus concentrations of TC in 0–8 µM; Figure S6: Recovery tests of NCDs@(4-Hap)_4_(Mo_8_O_26_) for TC sensing.
