# Supplementary material for: Nitrogen-Doped Carbon Dots Encapsulated a Polyoxomolybdate-Based Coordination Polymer as a Sensitive Platform for Trace Tetracycline Determination in Water
Source: Nanomaterials (Basel). 2023 Sep 29;13(19):2676. doi: 10.3390/nano13192676 (PMC10574045; doi:10.3390/nano13192676)
Supplement: Supplementary file 1 [file nanomaterials-13-02676-s001.zip › nanomaterials-2613811-supplementary.pdf]

Nitrogen-doped carbon dots encapsulated a polyoxomolybdate-based coordination polymer as a sensitive platform for trace tetracycline determination in water

Yanqiu Zhang[1,2,*], Minrui Sun[1], Yang Lu [1], Mingguo Peng[2,*], Erdeng Du[2], Xia Xu[2]

[1] School of Environmental Science and Engineering, Changzhou University, Changzhou 213164, PR China；sunmrcczu1998@163.com (MR.S.); m17625046190@163.com (Y.L.)

[2] School of Urban Construction, Changzhou University, Changzhou 213164, China, PR China；duerdeng@gmail.com (ED.D.); xuxia@cczu.edu.cn (X.X.)

* Correspondence: zhangyanqiu@cczu.edu.cn (YQ. Z.); pmg@cczu.edu.cn (MG. P.)

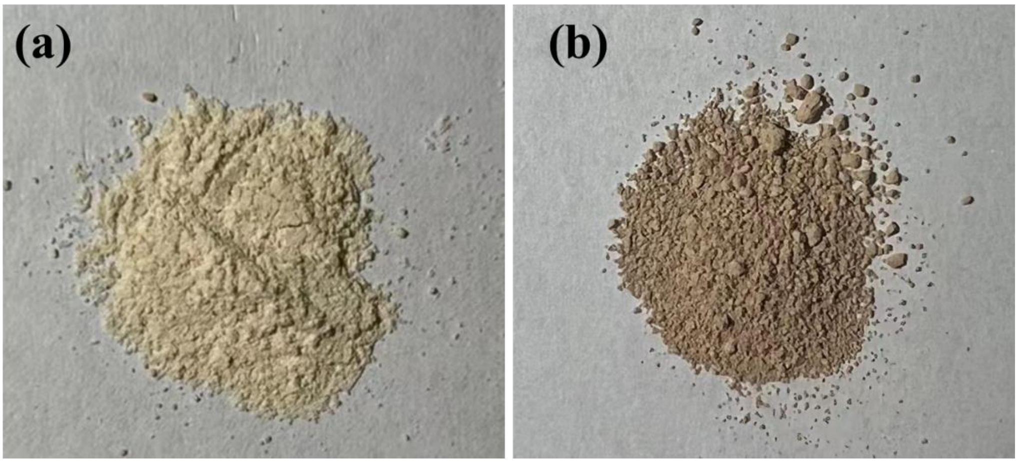

Figure S1. Photographs of (a) (4-Hap)$_4$(Mo$_8$O$_{26}$), (b) NCDs@(4-Hap)$_4$(Mo$_8$O$_{26}$) under natural light.

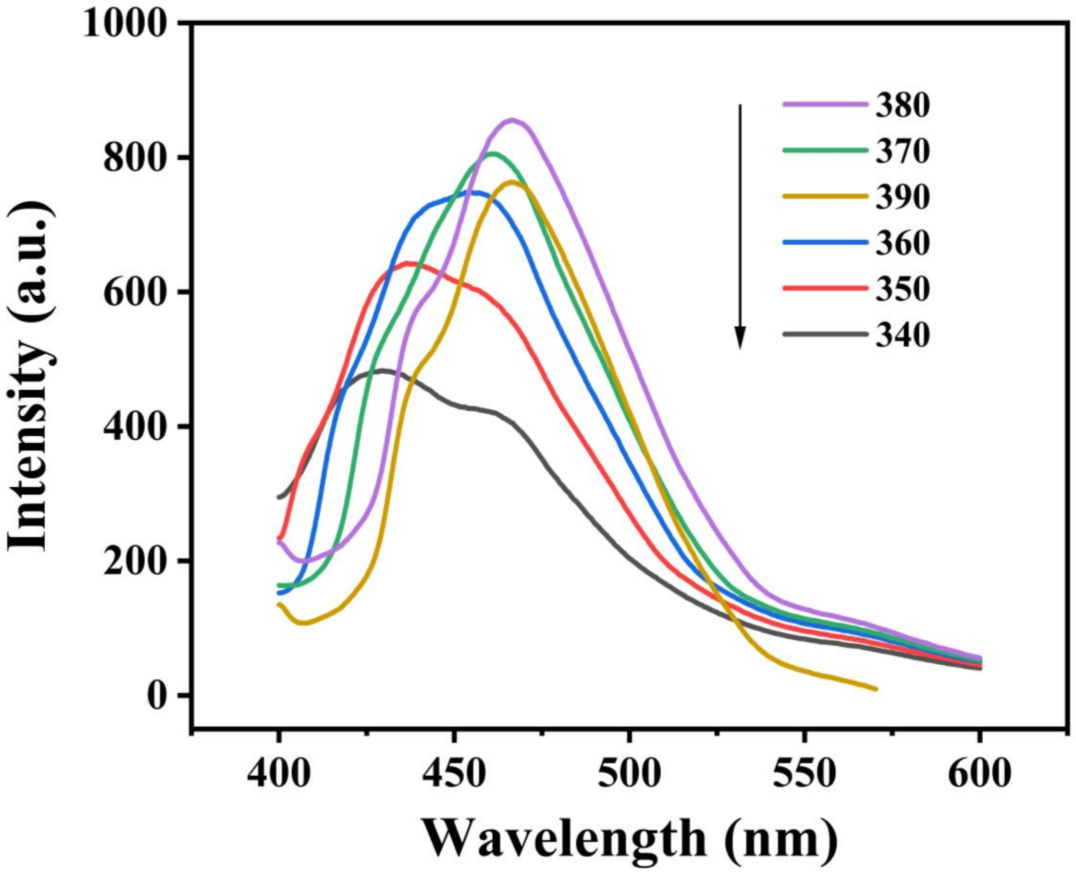

Figure S2. Emission spectra for NCDs@(4-Hap)$_4$(Mo_8O_{26}) in water at different wavelengths.

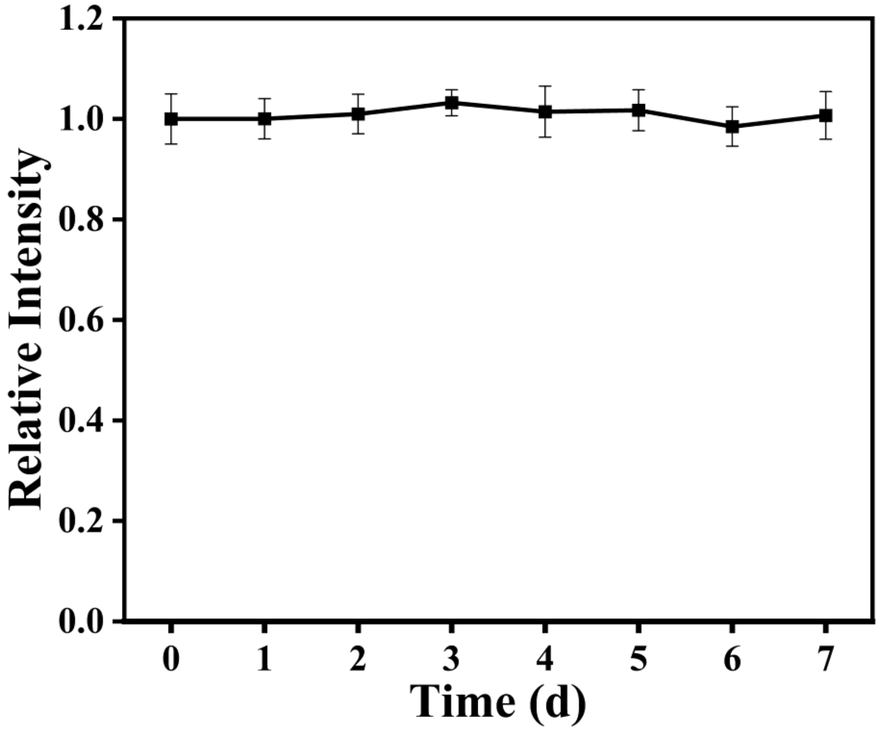

Figure S3. Relative fluorescence intensity of NCDs@(4-Hap)$_4$(Mo_8O_{26}) with the excitation at 380 nm in 7 days.

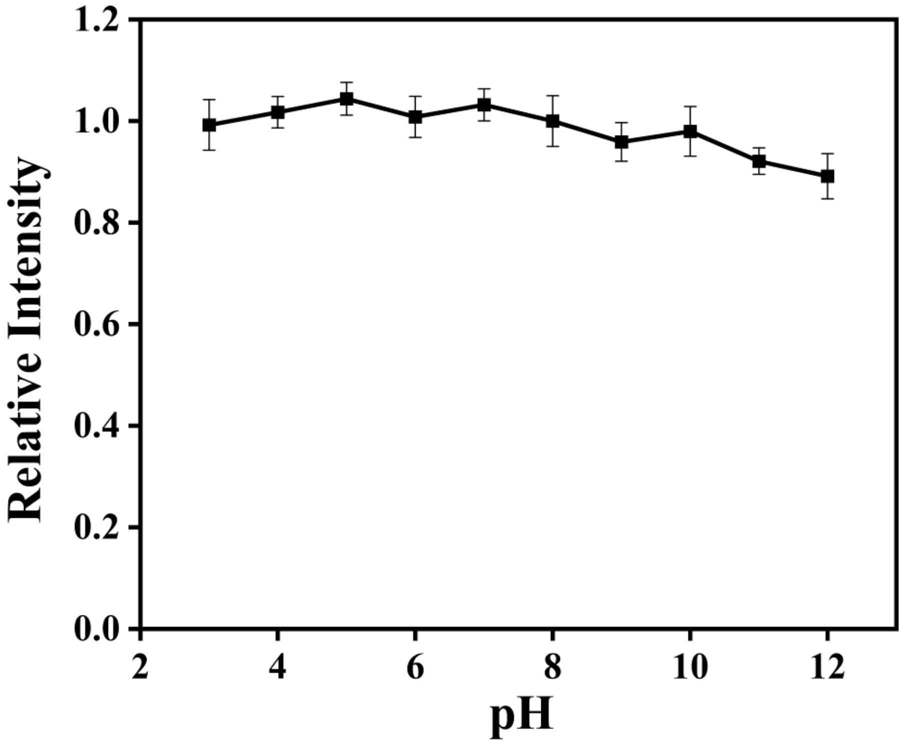

Figure S4. The relative fluorescence intensity of NCDs@(4-Hap)$_4$(Mo$_8$O$_{26}$) in water under different pH (E$_x$= 380 nm).

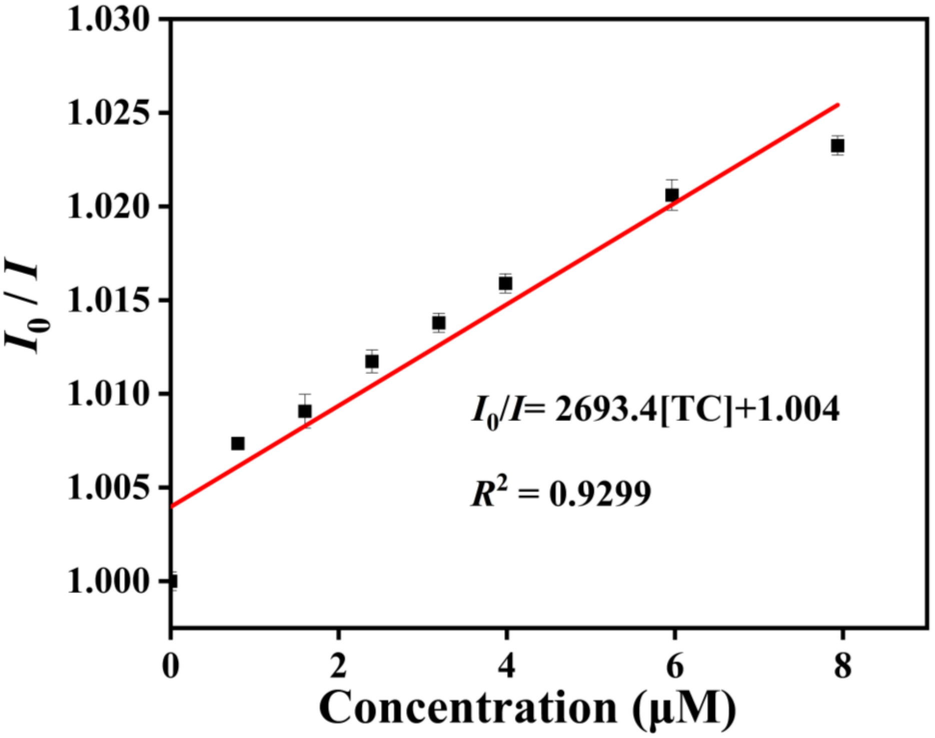

Figure S5. Calibration plot of the fluorescence intensity ratio (I_0/I) versus concentrations of TC in 0-8 μM.

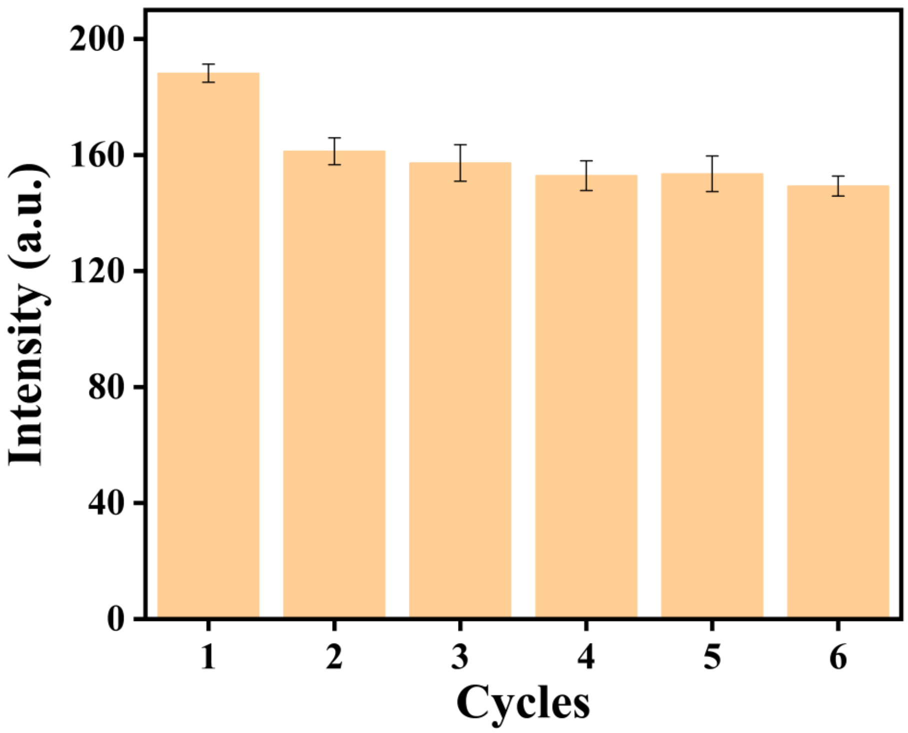

Figure S6. Recovery tests of NCDs@(4-Hap)$_4$(Mo_8O_{26}) for TC sensing.
